# 

**Published:** 1879-07

**Authors:** 


### Books and Periodicals.

We acknowledge the receipt of the following new publications :

*Physiology*: Preliminary Course of Lectures by Jas. T. Whittaker, M. A., M. D.; illustrated. C. R. Murry, publisher, Cincinnati, Ohio.

*Young Scientist*, a practical journal for Amateurs. Vol. II.; No. 3; price fifty cents a year; New York.

New and Original Theories of the Great Physical Forces, by H. R. Rogers, M. D., Dunkirk, N. Y. To be had of the author.

*New Preparations*, a monthly journal of Medicine, edited by Wm. Brodie, M. D. Geo. S. Davis, publisher, Detroit, Mich. Price one dollar a year.

*Urethrismus*, or Chronic Spasmodic Stricture, by F. N. Otis, M. D., Clinical Professor of Genito-Urinary Diseases in the College of Physicians and Surgeons, New York. Address the author.

*Quarantine*, its Sanitary and Political Aspect in relation to the spread of Epidemic Diseases, by J. C. Le Hardy, M. D., Savannah, Ga. Jas. P. Harrison & Co., publishers, Savannah.

*Index Medicus*, a monthly classified record of the current medical literature of the world. Compiled under the supervision of Dr. John S. Billings, Surgeon U. S. Army, and Dr. Robert Fletcher, M. R. C. S., England. Vol. I.; No. 4. New York: F. Leypoldt, 13 and 15 Park Row. $3.00 per year.

*Archives of Medicine*, a bi-monthly journal; edited by E. C. Sequin, M. D., New York. J. P. Putnam's Sons, 182 Fifth Avenue.

### SUBSCRIPTIONS RECEIVED

To July 1, 1879.

In this column we will credit every subscription received during the last quarter. Subscribers will please notify us immediately if their payments are not duly credited. The State only is given—this, with the full name of the party subscribing, will be sufficient to indicate who is meant:

NEW YORK—Dr M Besemer, S H Cotrell, Mrs Polly Caldwell, Mrs Dr Washington, Jno Woolworth, Theo B Perry, H A Best, Dr J B Sargent, H B Gilbert, Mrs C E Rapeiyea, Dr L Van Kleek, Dr F Jackel, Hon S H Ainsworth, G E Lawrence, E Robinson, Dr J H Hildreth, Dr H Slosson, Dr G Pratt, Mrs Emberlie, Dr L M Seymour, Dr J H Godfrey, Dr Lyman Smith, Dr P Elliott, Geo H Peters, Seymour Archer, Simeon Struble, Dr T Ames, Dr L Benjamin, Dr R B Emerson, Dr Gridley, Dr J I Snyder, Dr C Coe Dr P B Woodward, Dr L Irving, Dr B Bliss.

PENNSYLVANIA—A A Root, Wm Scott Dr D E Rice, Wm Stewart, Dr J F Kocher, Henry Wilson, Dr E P Luce, Dr P Heinmann, S Polk, H Shaw, R J Wisner, Dr V B Martin, Dr O Gutelius, Dr J Pennybaker, Dr J D Forbes, Dr P L Shindel' Dr J Freelingheisen, Dr D D Olive, Dr Simon Lown, Dr Chas Carpenter, Dr L L Ulrich, Dr Stephen Hyt, Dr Jas Johnson, Dr A Truman, Dr A Lafever, Dr O B Howe, Dr H Simons, Dr Wm Dwight, Dr H Fisher, Dr Sam Tillinghast Dr P Brown, Dr Jas Jackson, Dr Leverich, Dr DeWitt Groom, Dr Wiliam Samuels, Dr J I Attley, Dr F Cummings, Mrs A B Smith, John T Wythe, Geo L White, Salmon Riggles, A B Wythe, Dr Irvington.

CONNECTICUT—E L Marvin, Dr Samuel Green, Dr O O White, Mrs. J B Parsons, Irving Bishop, Dr Chas Ingraham.

COLORADO—Dr W B Sutherland, Mrs Geo H Wharton.

DIST COL—A Zoller, J H Norton, A Bishop, Dr A B Williamson, Dr L Barnes.

FLORIDA—Dr J Harkinson, Dr D D La Montenyia, Dr John Oglesbee, Mrs Sampron, Geo H Livermore.

GEORGIA—Dr B N Bureguard, Dr Richard LaTrobe.

ILLINOIS—Dr Knox Jessup, Dr B G Tice Dr L Battey.

MICHIGAN—Dr L W Farquelle, Dr S DuBois, A G Weston, Dr D D Platt.

MISSOURI—Dr J S Wallace, Dr S N Snow.

NORTH CAROLINA—G B Smith.

OHIO—John A Novinger, Mrs Wm Savage, Dr Chas Sampson.

TEXAS—Dr Jas R Mills, Dr A Bengall.

OFFICE PAYMENTS—American News Company, Pacific Subscription Agency.

